# Identification of potent anti-*Candida* metabolites produced by the soft coral associated *Streptomyces* sp. HC14 using chemoinformatics

**DOI:** 10.1038/s41598-023-39568-7

**Published:** 2023-08-02

**Authors:** Bahaa Abdella, Mohamed Abdella, Hafed A. ElSharif, Amani M. D. ElAhwany, Nermeen A. El‑Sersy, Hanan A. Ghozlan, Soraya A. Sabry

**Affiliations:** 1 Faculty of Aquatic and Fisheries Sciences, Kafrelsheikh University, Kafrelsheikh, 33516 Egypt; 2 Botany and Microbiology Department, Faculty of Science, Alexandria University, Alexandria, 21511 Egypt; 3 Marine Microbiology Laboratory, National Institute of Oceanography and Fisheries, NIOF, Alexandria, Egypt; 4 Present Address: Department of Botany, Faculty of Arts and Sciences, University of Benghazi, Benghazi, Libya

**Keywords:** Microbiology, Bacteriology

## Abstract

*Candida albicans* is the most common pathogen responsible for both spontaneous and recurrent candidiasis. The available treatment of *Candida* infections has several adverse effects, and the development of new drugs is critical. The current study looked at the synthesis of anti-*Candida* metabolites by *Streptomyces* sp. HC14 recovered from a soft coral. Using the Plackett Burman design, the medium composition was formulated to maximize production. Using GC–MS, the compounds have been identified, and a cheminformatics approach has been used to identify the potential source of activity. The compounds that showed high potential for activity were identified as pyrrolo[1,2-a]pyrazine-1,4-dione, hexahydro-3-(phenylmethyl)-3 and di-n-octyl based on their docking score against the cytochrome monooxygenase (CYP51) enzyme in *Candida albicans*. As a result of their discovery, fewer molecules need to be chemically synthesized, and fermentation optimization maximizes their synthesis, providing a strong foundation for the development of novel anti-*Candida albicans* agents.

## Introduction

*Candida albicans* is normal flora of the oral cavity, accounting for up to 75% of the microbial population^1^. Approximately 70% of humans have this opportunistic fungal infection as a harmless commensal in their genitourinary and gastrointestinal tracts^2^. On the other hand, with mortality rates reaching up to 50%, *Candida* spp. is one of the most dominant causes of hospital-acquired systemic infections in the United States^3,4^. *Candida albicans* infection of the oral cavity or female genitalia causes superficial candidiasis, while systemic infections are the most dangerous type of infection^1^. In the systematic infections the heart, blood, bones, eyes and brain might get affected^5^. For vaginal candidiasis, between 85 and 95% of the yeast strains isolated from the vagina are *C. albicans* strains^6^. *Candida* infection begins under certain conditions, such in diabetic individuals, during pregnancy, and when taking antibiotics^6^. To treat *Candida* spp. infections, a limited class of synthetic drugs such as ketoconazole, nystatin, and fluconazole have been produced; nevertheless, misuse of such compounds may exert a selection pressure toward resistance^7^. Given the adverse effects of these synthetic chemicals, it is critical to constantly supply the market with new natural compounds that could be used as alternatives to such substances. Microbial diversity is abundant in aquatic habitats, and microorganisms are associated with other multicellular aquatic creatures^8–11^. Because of the unique physicochemical characteristics obtained through living in extreme conditions, marine organisms have attracted the attention of researchers^12^. Antibiotics are thought to be primarily produced by actinomycetes. In the form of primary or secondary metabolites, it has thus always been a vital source of inspiration for medicinal chemists^13,14^. Marine actinomycetes have been isolated from marine sediments and have been linked to marine animals such as sponges^15^. The production of bioactive compounds by actinomycetes and other microorganism is influenced by organism’s genotype, metabolism, physiology, and fermentation conditions^16^. External parameters such as inoculation size, medium volume, incubation period, incubation temperature, agitation speed, and starting pH have all had a significant impact on the nature and quantity of antibiotics and/or bioactive chemicals produced^17–20^. In the field of antibiotics, much effort was invested in directing the product range and enhancing production rates. Changing nutritional or environmental conditions has been demonstrated to result in the development or discovery of novel natural chemicals utilizing various approaches^21^. However, the poor yield of these chemicals limits their application in many circumstances. Furthermore, the nutritional requirements of the microbes used in industrial fermentation processes are as complex and diverse as the bacteria themselves^22,23^. To that end, and thanks to advances in computational biology, drug discovery and repurposing of natural microbial compounds have become easier and faster^24^.

In the current study, the most influential factor for *Streptomyces* sp. HC14 anti-*Candida* activity was examined using the Plackett–Burman design. The generated compounds were identified using gas chromatography-mass spectrometry (GC–MS) under near-optimal conditions. The putative active compounds were computationally investigated against a fundamental protein target in *Candida albicans* utilizing a molecule docking and molecular dynamics approach.

## Materials and methods

### Bacterial strain and growth condition

The actinobacteria *Streptomyces* sp. HC14 was previously isolated from the soft coral *Sarcophyton glaucum* that has been collected from the Red Sea, Hurghada, Egypt^25^. The 16 s rRNA sequence of *Streptomyces* sp.HC14 was deposited in the NCBI GenBank under the accession numbers JQ929066^25^. For cultivation, a loopful of *Streptomyces* sp. HC14 was inoculated into 50 mL of ISP2 medium (Difco) in 250 mL Erlenmeyer flasks. Then it was incubated at 37 °C under shaking condition at 200 rpm for 7 days. The cell-free medium filtrate was used to test the antimicrobial activity of the fermented medium. The ISP2 medium has a composition of (g/L) yeast, 4; malt extract, 10; dextrose, 4. The medium pH was adjusted to pH 7.2.

### Anti-*Candida albicans* activity

The cell-free supernatant was used to test the antimicrobial activity of the tested strain. Cells were removed by centrifugation at 5000 rpm for 15 min. Fifty microliters of the *Streptomyces* sp. HC14 cell-free supernatant were tested against *Candida albicans* using the well diffusion method. After dispensing of the 50 µL to each the wells, plates were put in the refrigerator for 3 h to allow diffusion, then all the plates were incubated at 37ºC and diameter of inhibition zones were measured after 48 h^26^, and the well diameter was subtracted from the inhibition zone diameter.

### Plackett–Burman experimental design (PB)

*Streptomyces* sp. HC14 were potent in producing antagonistic compounds to *Candida albicans*. Therefore, it was selected for the optimization experiments in an attempt to maximize the production. Optimization was performed by a two-phase experimental design: the first is screening for critical elements that influence the production of antimicrobial agents in shaken flasks using Plackett–Burman design^27^, and the second was the verification experiment, to confirm the near-optimal conditions for production of anti-*Candida* compounds.

Seven independent variables based on the ISP2 medium’s composition were chosen for screening the most influential component. Namely, dextrose, yeast extract, malt extract, seawater, pH, culture volume, and inoculum size. For each variable, high ( +) and low (-) levels were tested (Tables 1, 2). Trial number 9 in Table 2 represents the basal control. All experiments were carried out in duplicate, and the arithmetic means of antibacterial activity were assessed by measuring the inhibition diameter as a response. The main effect of each variable was determined by the following equation:

$${\text{E}}_{{{\text{xi}}}} = \left( {\sum {\text{M}}_{{{\text{i}} + }} - \sum {\text{M}}_{{{\text{i}} - }} } \right)/{\text{N}}$$

where E_xi_ is the variable main effect, M_i+_ and M_i−_ are inhibition zone diameters in high and low levels respectively, where the independent variable (xi) was present in high and low concentrations, respectively, and N is the number of trials divided by 2. Statistical *t*-values for equal unpaired samples were calculated using Microsoft Excel to determine the variable significance. From the main effect calculations, the composition of the near optimized medium was predicated, which gives a maximum inhibition zone as a response.

**Table 1:** Screening of factors affecting the antimicrobial activity of *Streptomyces* sp. HC14 and their levels in the Plackett–Burman experiment design.

Factors	Symbols	Levels		
		High level (+)	Basal medium 0	Low level (−)
Yeast extract (g/L)	Ye	6	4	2
Dextrose (g/L)	Dx	6	4	2
Malt extract (g/L)	Ma	15	10	5
Sea water (%)	S.W	100% + 5 g/L NaCl	100%	50%
pH	pH	7.5	7	6.5
Culture volume (mL)	C.V	75	50	25
Inoculum size (mL)	I.S	1.5	1	0.5

**Table 2:** The applied Plackett–Burman experimental design for 7 culture variables.

Trials	Independent variables						
	pH	I.S	C.V	S.W	Ma	Dx	Ye
1	−	+	+	+	−	−	−
2	+	+	−	−	−	−	+
3	+	−	+	−	−	+	−
4	−	−	−	+	−	+	+
5	+	−	−	+	+	−	−
6	−	−	+	−	+	−	+
7	−	+	−	−	+	+	−
8	+	+	+	+	+	+	+
9	0	0	0	0	0	0	0

### Extraction of secondary metabolites

At the end of the fermentation period, the bioactive compounds were extracted from the culture medium using the method described by El-Naggar et al.^28^. Briefly, the cell-free extract was obtained by centrifugation for 15 min at 5000 rpm, then, it was mixed with chloroform in a 1:1 ratio and agitated for one hour and repeated 3 times. The organic phase was separated from the aqueous phase using a separation funnel and dried under a vacuum using a rotary evaporator at a temperature of no more than 50 °C. The recovered crude extract was then dissolved in methanol for further characterization.

### Structural and molecular docking

#### Analysis by UV absorption and GC–MS

The crude extract was analyzed by UV absorption using a Perkin Elmer–lambada 4B-UV/VIS Spectrophotometer. Identification of the metabolites was done by GC–MS analysis; briefly, 1 μL of the crude extract was injected into RTX-5 column (7 m × 0.32 mm) (model GC-MS-QP-2010 plus from Shimadzu, Japan), and Helium (3 mL/min) was used as a carrier gas. The following temperature gradient program was used: At 75 °C for 2 min, followed by an increase from 75 to 175 °C at a rate of 50 °C per min, and finally 7 min at 175 °C. The m/z peaks, representing mass to charge ratios characteristic of the metabolites, were compared with those in the mass spectrum library of the corresponding organic compounds^29^.

#### Ligand and protein model and docking

*Candida* target was retrieved from the PDB with the accession number 5V5Z. MarvinSketch (v22.11.0) was used to draw all the metabolites (ligands) 3D structures of the identified compounds. The ligands and protein model preparation and docking were conducted using Schrödinger software, released 2018-4^30,31^. Briefly, the drawn 3D structures were prepared using LigPrep tool. OPLS-2005 force field module was applied to minimize the energy of the ligands. The energy of each molecule was minimized using LigPrep. The docking was done using extra-precision docking default settings and the docking score results were displayed using XP visualizer.

### Ligand-target complex free energy

To calculate the free energy of complexes, Maestro’s Prime integrated tool was used. Molecular mechanics of the Generalized Born and surface area solvation (MM-GBSA) method was used to determine the binding free energies^32^. MM-GBSA was used to improve the accuracy of the docking score^33^. Thus, the free energy of optimized receptors, ligands, and ligand-receptor complexes was calculated. These calculations were performed under the solvation condition of the VSGB 2.0 model and forcefield OPLS_2005^34^. The strain energy for the ligands as well as the relative binding free energies of the complexes were also estimated^34,35^. The visualization of energy was generated by the primary energy visualizer^36^.

### Ethics approval

This article does not contain any studies on humans or on animals.

## Results

### Plackett–Burman experimental design (PB)

Plackett–Burman design was used in a few controlled experiments to determine the factors affecting the production of bioactive chemicals and figure out their likely ideal amounts. The responses in Table 3 demonstrate a significant variation in the diameter of the inhibition zones, highlighting the significance of parameter tuning to achieve high activity. This illustrates that the combined influence of the nine components in their inhibition zone diameter range from 0 to 15 mm against *Candida albicans*.

**Table 3:** Plackett–Burman experimental design for seven variables and the corresponding responses for evaluating factors influencing the production of *Streptomyces* sp. HC14 bioactive compounds against *Candida albicans*.

Trials	Factors							Inhibition zone (mm)
	Ye	Dx	Ma	S.W	C.V	I.S	pH	
1	−	−	−	+	+	+	−	0
2	+	−	−	−	−	+	+	5
3	−	+	−	−	+	−	+	7
4	+	+	−	+	−	−	−	15
5	−	−	+	+	−	−	+	0
6	+	−	+	−	+	−	−	0
7	−	+	+	−	−	+	−	0
8	+	+	+	+	+	+	+	0
9	0	0	0	0	0	0	0	8

**Figure 1:**
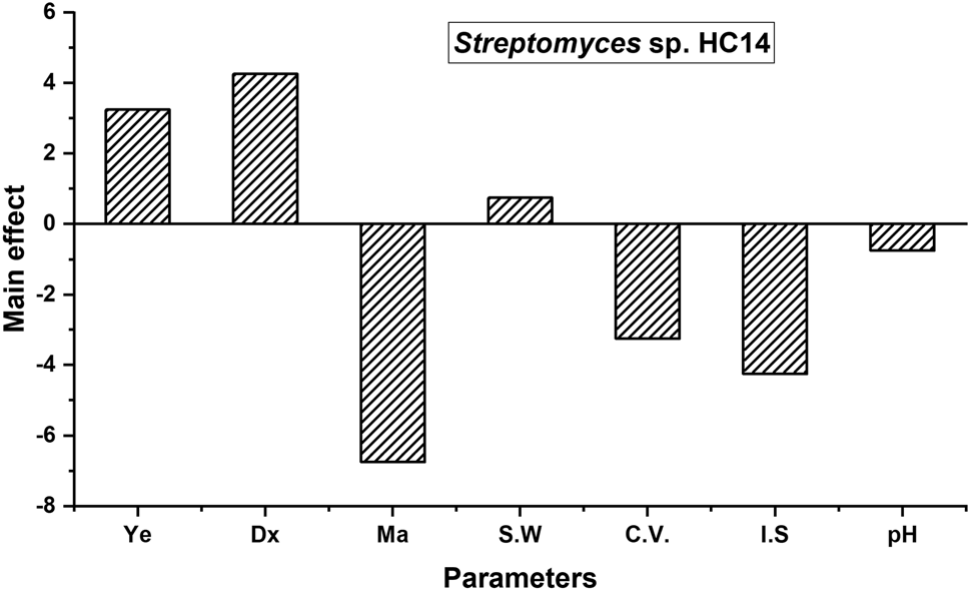
Positive and negative influences of the studied variables on bioactive compound production by *Streptomyces* sp HC14 based on Plackett–Burman results.

The main effect of each variable was estimated as the difference between the average of the measurements made at the high (+) and low (−) levels of the factors. It was observed for antagonistic compounds produced by *Streptomyces* sp. HC14 for *C. albicans*. High levels of dextrose, sea water, and yeast extract enhanced anti yeast activity. On the contrary, low levels of malt extract, pH, volume, and inoculum size increased the zone of inhibition (Fig. 1).

Statistical analysis of the Plackett–Burman experiment is shown in Table 4 for *Streptomyces* sp. HC14. From a closer look, malt extract was the most significant variable for *Streptomyces* sp. HC14, as it had significant levels of 95%.

**Table 4:** Statistical analysis of the Plackett–Burman experiment results for *Streptomyces* sp. HC14.

Factor	Main effect	t-test	*p* value	Significance (%)
Yeast extract (g/L)	3.25	0.83	0.22	78
Dextrose (g/L)	4.25	1.12	0.16	84
Malt extract (g/L)	− 6.75	− 2.16	0.05	95
Sea water	0.75	0.18	0.43	57
pH	− 0.75	− 0.18	0.43	57
Culture volume	− 3.25	− 0.82	0.22	78
Inoculum size	− 1.75	− 1.12	0.16	84

### Verification experiment

**Figure 2:**
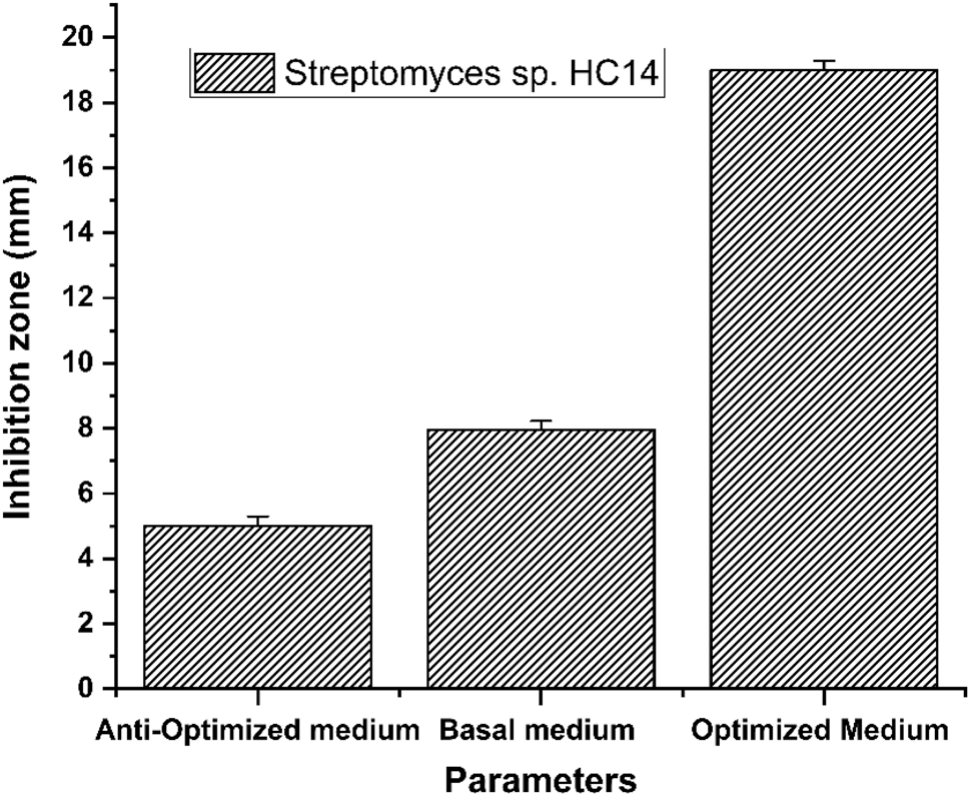
Verification experiments of the applied Plackett–Burman statistical design by comparing the inhibition zone of the cell-free culture medium of *Streptomyces* sp. HC14 against *Candida albicans*. *Streptomyces* sp. HC14 was grown on ISP2 medium (basal medium), optimized, and anti-optimized media. Error bars represent the SEM (n = 3).

To validate the obtained data and to evaluate the accuracy of the applied Plackett–Burman statistical design, a verification experiment was carried out in triplicate for each strain to predict the near optimum combination levels of independent variables. The Plackett–Burman design predicted that the higher inhibition zone formed by *Streptomyces* sp. HC14 was grown in a medium containing (g/L): yeast extract 6, Dextrose 6, Malt extract 5, Sea Water + 5 g NaCl, pH 6.5 with an inoculum size of 0.5 mL in 25 mL culture volume. Three independent repeated experiments were performed to verify the validity of the near optimum settings. The base condition was compared to the average inhibition zone of anticipated near-optimal levels of independent variables. Using the optimal conditions established from the Plackett–Burman experiment, the inhibition zone of *Streptomyces* sp. HC14 rose 2.37 folds over the basal conditions (Fig. 2).

### Structural and molecular docking

#### Identification of secondary metabolites

**Figure 3:**
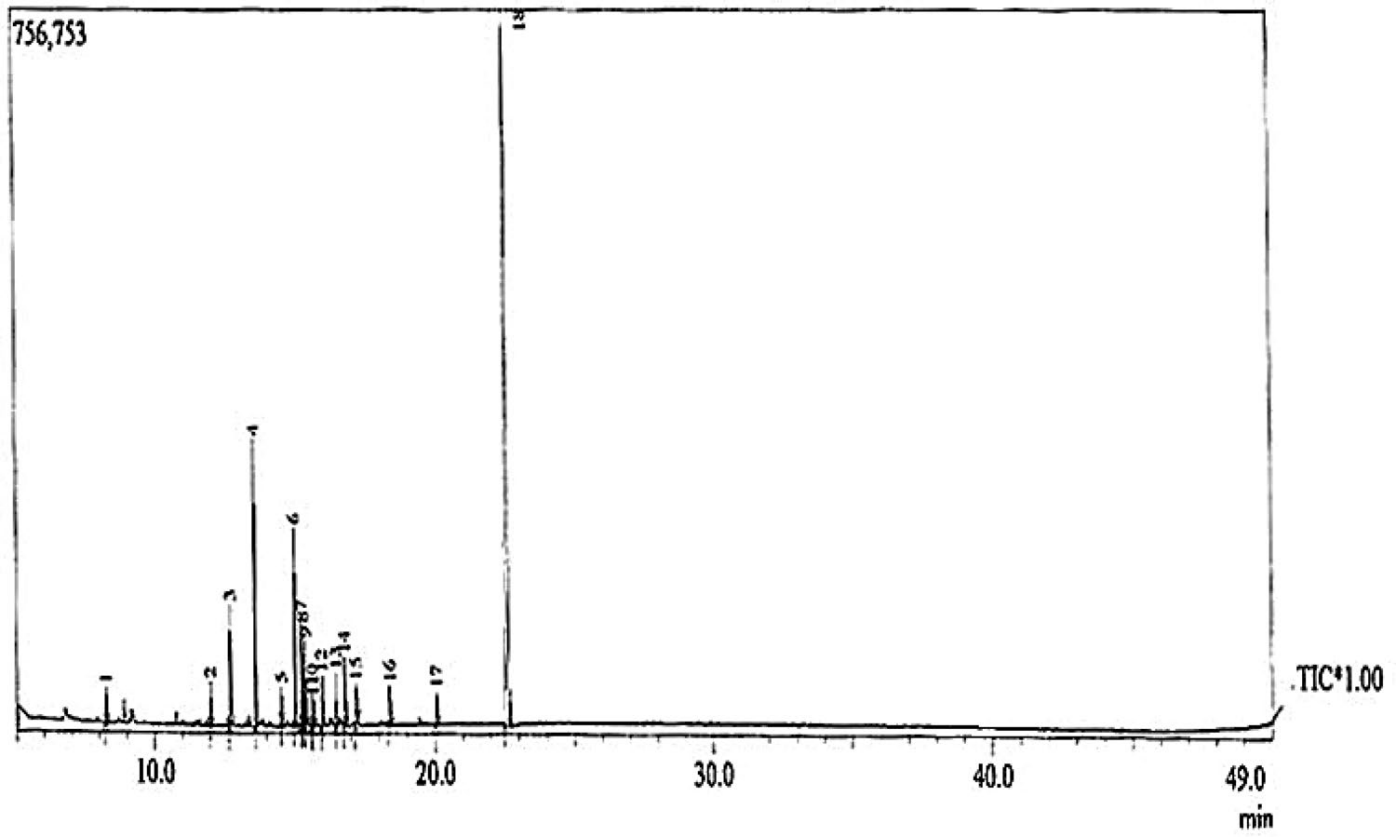
GC Mass Chromatogram analysis for purified compounds extracted from *Streptomyces* sp HC14.

For the identification of secondary metabolites, UV absorption followed by GC–MS were applied to the active crude extract. The UV absorption spectrum of the extract with antimicrobial activity has recorded a maximum absorption at 437.4 nm for *Streptomyces* sp. HC14. The National Institute of Standard and Technology (NIST) database, which contains more than 62,000 patterns, was used to interpret the mass spectrum of the GC–MS. A comparison was made between the mass spectra of the unknown and known components recorded in the NIST collection. The components of the test materials’ names, molecular weights, and structures were determined. The GC–MS chromatograms of *Streptomyces* sp. HC14 metabolites is given Fig. 3. On comparison of the mass spectra of the constituents with the NIST library, eighteen peaks were obtained for *Streptomyces* sp. HC14, (Table 5). As clearly shown in the Fig. 3 the highest peak was displayed by the 18th compounds which has been identified to be di-n-octyl phthalate. The second highest peak was compound ordered the 4th, named Cyclohexane, 1, 5-diisopropyl-2, 3-dimethyl.

**Table 5:** *Streptomyces* sp. HC14 metabolites present in the anti-*Candida* active conditioned medium.

ID	Metabolite
1	Maltol
2	1-Hexene, 2, 4, 4-triethyl
3	Diethyl phthalate
4	Cyclohexane, 1, 5-diisopropyl-2, 3-dimethyl
5	2-Decene, 3-methyl-, (Z)
6	Hexadecanoic acid, methyl ester
7	N- Hexadecanoic acid
8	Pyrrolo[1, 2-a]pyrazine-1, 4-dione, hexahydro-3-(2-methylpropyl)
9	1, 2-Benzenedicarboxylic acid, bis (2-methylpropyl)ester
10	Heptane, 1-iodo-
11	Pentanol, 2, 2, 4-trimethyl
12	4-Bromoheptane
13	Octadecanoic acid, methyl ester
14	Nonadecanoic acid
15	DL-Leucine, N-DL-leucyl-
16	Oxirane (tetraadecyloxy) methyl
17	Pyrrolo[1, 2-a]pyrazine-1, 4-dione, hexahydro-3-(phenylmethyl)
18	Di-n-octyl phthalate

#### Molecular docking

**Figure 4:**
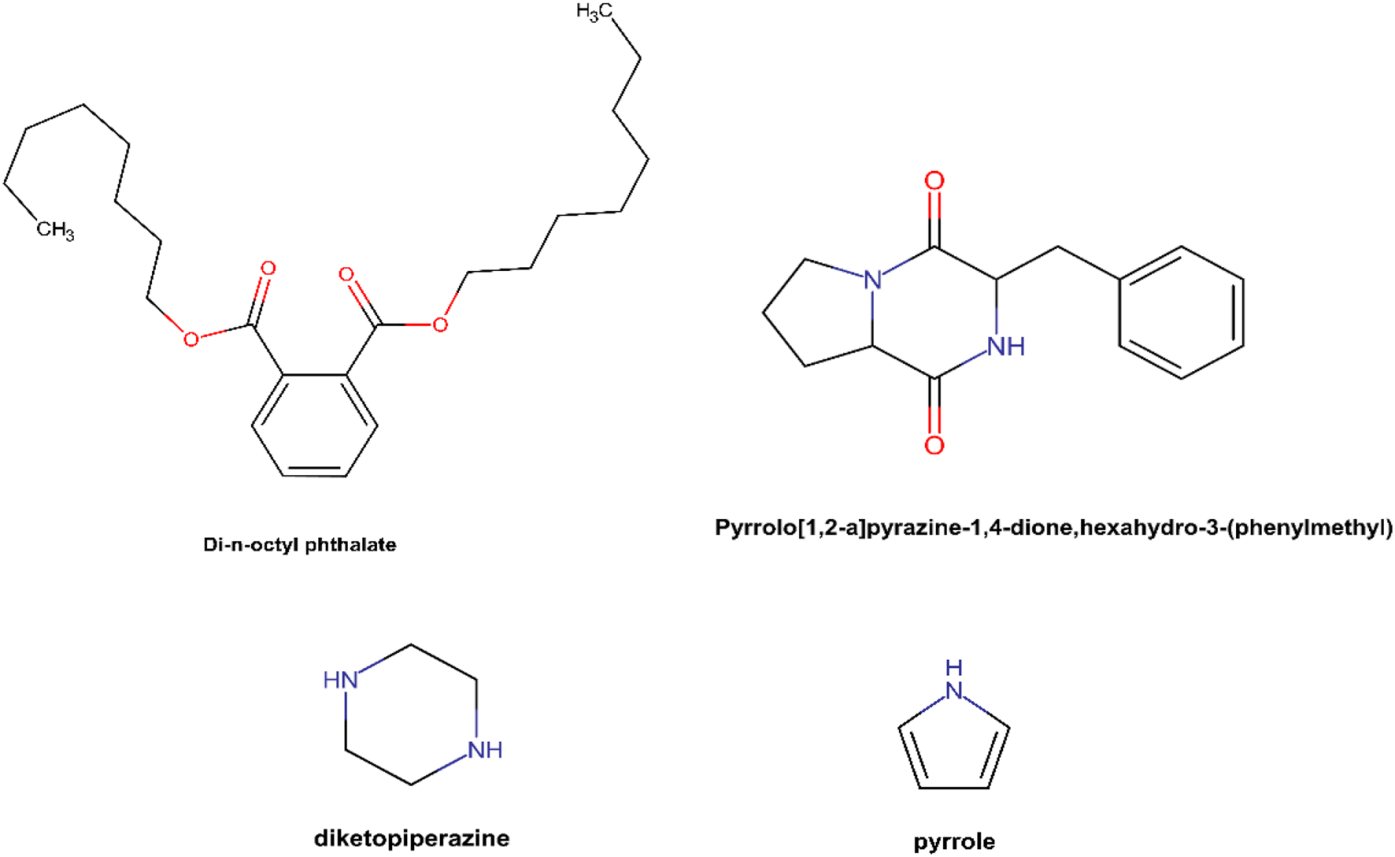
(**A**) Di-n-octyl phthalate, (**B**) Pyrrolo[1,2-a]pyrazine-1,4-dione,hexahydro-3-(phenylmethyl) and its precursors, (**C**) Diketopiperazine, (**D**) Pyrrole.

To investigate the potential interaction between the identified metabolites and the target protein , molecular docking techniques were employed. Molecular docking was carried out between 18 compounds and CYP51 enzyme in *Candida albicans*. The docking results (kcal/mol) of the extract’s bioactive compounds of *Streptomyces* sp. HC14 against *Candida albicans* CYP51 enzyme are shown in Table 6. The main purpose of this step was to explore and predict the source of activity in the extract and the potential target. From the results listed in Table 6, it was observed that pyrrolo[1, 2-a]pyrazine-1, 4-dione, hexahydro-3-(phenylmethyl) showed the best scores for docking, GScore, lipophilic score and H-Bond energy with values of − 10.428 kcal/mol, − 10.428 kcal/mol, − 3.747 kcal/mol, and − 1.725 kcal/mol respectively. Figure 4 depicts the chemical structures of the top metabolites based on docking score.

**Table 6:** Molecular docking scores (kcal/mol) of identified compounds of *Streptomyces* sp. HC14 active metabolites against CYP51 enzyme (PDB: 5V5Z) from *Candida albicans*.

Rank	Metabolite	Docking score (kcal/mol)	GScore (kcal/mol)	Lipophilic EvdW (kcal/mol)	H-Bond (kcal/mol)
1	Pyrrolo[1, 2-a]pyrazine-1, 4-dione, hexahydro-3-(phenylmethyl)	− 10.428	− 10.428	− 3.747	− 1.725
2	Di-n-octyl phthalate	− 8.299	− 8.299	− 7.119	0
3	Pyrrolo[1, 2-a]pyrazine-1, 4-dione, hexahydro-3-(2-methylpropyl)	− 8.193	− 8.193	− 3.313	− 0.7
4	DL-Leucine, N-DL-leucyl-	− 6.96	− 7.023	− 3.001	− 2.049
5	Cyclohexane, 1, 5-diisopropyl-2, 3-dimethyl	− 6.822	− 6.822	− 4.188	0
6	Nonadecanoic acid	− 6.241	− 6.244	− 4.526	− 1.552
7	n-Hexadecanoic acid	− 5.897	− 5.901	− 4.016	− 2.09
8	Octadecanoic acid, methyl ester	− 5.686	− 5.686	− 5.358	− 0.984
9	Pentanol, 2, 2, 4-trimethyl	− 5.53	− 5.53	− 2.481	− 1.395
10	1,2-Benzenedicarboxylic acid, bis(2-methylpropyl) ester	− 5.49	− 5.49	− 4.555	0
11	Maltol	− 5.33	− 5.364	− 2.2	− 1.397
12	Diethyl phthalate	− 5.102	− 5.102	− 3.055	− 1.109
13	Hexadecanoic acid, methyl ester	− 4.804	− 4.804	− 5.047	− 0.648
14	((Tridecyloxy)methyl)oxirane	− 4.567	− 4.567	− 4.855	− 1.059
15	1-Hexene-2, 4, 4-triethyl	− 4.34	− 4.34	− 3.541	0
16	2-Decene, 3-methyl-, (Z)	− 3.981	− 3.981	− 3.422	0
17	4-Bromoheptane	− 3.934	− 3.934	− 2.907	0
18	Heptane, 1-iodo-	− 2.990	− 2.990	− 2.900	0

**Figure 5:**
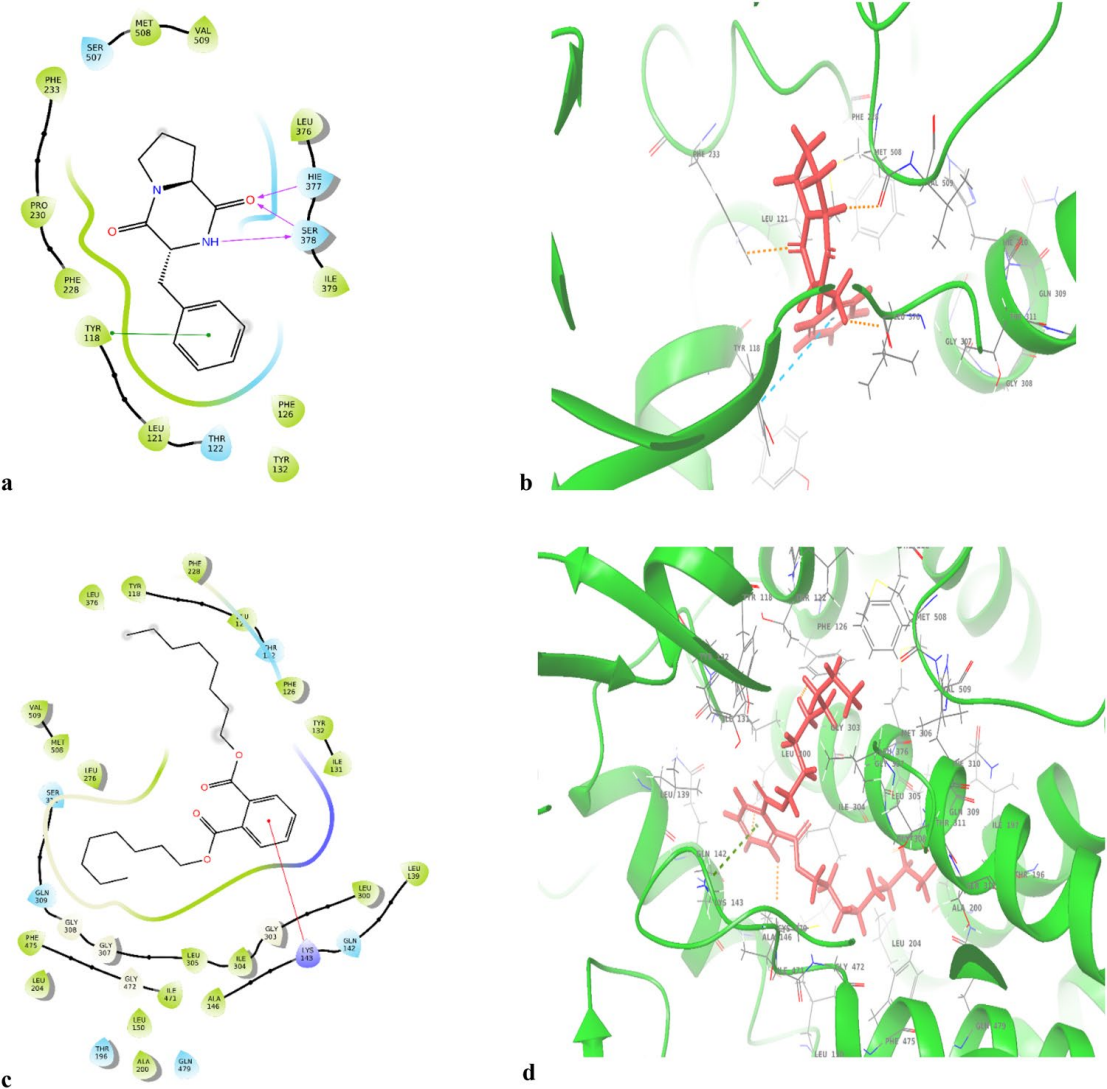
Pyrrolo[1, 2-a]pyrazine-1, 4-dione, hexahydro-3-(phenylmethyl) and di-n-octyl phthalate. 2D (**a**,**c**) and 3D (**b**,**d**) Docking complex of two compounds against CYP51 enzyme. Figures were generated using maestro ligand interaction diagram (2D) and Maestro interface (3D).

The graphical representations of the interactions in ligand-target complex are shown in Fig. 5a,b. The figures depict that there are three hydrogen bonds formed between the ligand and the SER-378 and HIE-377 amino acid residues of the target protein. Moreover, this metabolite showed one π-π with TYR-118 residue.

The second-best compound was di-n-octyl phthalate as shown in Table 6, it displayed values of − 8.299 kcal/mol, − 8.299 kcal/mol, and − 7.119 kcal/mol of dock score, GScore and lipophilic score, respectively. The interaction in ligand-target complex (Fig. 5c,d) showed 1 π-cation bond with LYS-143.

Prime MM-GBSA results in Table 7 revealed that the calculated binding energy of di-n-octyl phthalate-1 was − 80.92 kcal/mol which is highest among the tested compounds as well as it is higher compared to the best docking compound (pyrrolo[1, 2-a]pyrazine-1, 4-dione, hexahydro-3-(phenylmethyl)-3), which has a numerical values calculated to be − 54.51 kcal/mol. This indicates that di-n-octyl phthalate has a higher affinity to CYP51 enzyme.

**Table 7:** The relative binding free energies obtained by Prime MM-GBSA-free energies.

Metabolite	MM-GBSA dG bind	MM-GBSA dG bind coulomb	MM-GBSA dG bind(NS)	MM-GBSA dG bind(NS) coulomb
Pyrrolo[1, 2-a]pyrazine-1, 4-dione, hexahydro-3-(phenylmethyl)-3	− 54.51	− 18.12	− 58.57	− 17.43
Di-n-octyl phthalate-1	− 80.92	− 4.48	− 98.46	− 5.12

## Discussion

In the current study, the metabolites of *Streptomyces* sp. HC14 were computationally evaluated to predict likely active compounds from a mixture of metabolites that demonstrated activity against *Candida albicans*. The Plackett–Burman screening stage revealed that the culture conditions had a significant impact on the production of active chemicals. Similar observation was found during the optimization of anti-*Klebsiella* compounds production by *Streptomyces* sp. 2A^37^. Similar trend was also observed by *Streptomyces albus* AN1 and J1074^38^. The coherence occurred between *Streptomyces* sp. 2A and *Streptomyces* sp. HC14 in such a way that certain Plackett–Burman trials resulted in the entire absence of activity, indicating that the growing conditions are extremely important. From main effect calculation and analysis, malt extract was crucial component in the synthesis of anti-*Candida* metabolites, this was agreed with results the production of anti-*Agrobacterium tumefaciens* from *Streptomyces* sp. TN71^39^. With respect to concentration, the best concentration of malt extract was 5 g/L in the current study. The same concentration was reported for *Streptomyces* sp. TN71^39^. Smaoui et al., on the other hand, reported that a low concentration of glucose (2 g/L) was advantageous to the antimicrobial activity, whereas the highest concentration of glucose was helpful in the current study. Glucose and malt extract, together with other medium components, were crucial for enhancing the anti-fouling agent produced by *Streptomyces sampsonii* PM33^40^. Furthermore, malt extract was particularly beneficial in boosting up to 10.5-fold the Ilamycin-E1/E2 from *Streptomyces atratus* SCSIO ZH16-ilaR mutant, which is active against *Mycobacterium* TB^41^. Subsequently, the GC–MS analysis identified the composition of the metabolite, which, when combined with molecular docking, offered some new insight on the possible function of such molecules. Among the identified compounds, the concentration of di-n-octyl phthalate was the highest. It also resulted in high docking and MM-GBSA scores, which support the argument to be the main source of the activity of *Streptomyces* sp. HC14 extracts against *Candida albicans*. The activity is supported by the results reported by Shafeian et al.^42^, the compound di-n-octyl phthalate was purified from the marine sponge *Haliclona (Soestella) caerulea* and proved to be effective against the *Candida albicans*. Additionally, it was also isolated from sources for instance, *Streptomyces melanosporofaciens* and *Streptomyces albidoflavus*, *Penicillium skrjabinii*, and *Penicillium olsonii*^43^. It has been also identified in plant extract of *Sisymbrium irio* and exhibited antifungal activity^44^. This demonstrates the distribution of this molecule and demonstrates its promise as an antifungal drug, as well as supporting its activity and explaining the high affinity scores provided by cheminformatics.

The second potential active compound with high docking results was [1,2-a]pyrazine-1,4-dione, hexahydro-3-(phenylmethyl) which might have a role in the anti-*Candida* activity. The strong docking score supports it as a source of action and is consistent with prior studies, as it was isolated from *Streptomyces* sp. VITPK9 demonstrated anti-*Candida* activity with low MIC values. The MIC values ranged from 0.78 to 1.6 µg/mL against *Candida krusei* MTCC9215, *Candida tropicalis* MTCC184, and *C. albicans* MTCC227^45^. This backs with previous research on its antimicrobial/anti-*Candida* capabilities^45,46^.

The molecular backbone is made up of diketopiperazine and pyrrole. The diketopiperazine and its derivatives were found to have antifungal properties, such as cyclo(L-Ile-L-Pro) and cyclo(Gly-Leu), which were isolated from Gram-negative bacteria such as *Pseudomonas aeruginosa* and *Lactobacillus plantarum*, respectively^47^. Furthermore, pyrrole has been discovered to be a high potential chemical with intriguing applications that is efficacious against multidrug resistant bacteria^46^. In other words, it has maintained the antifungal activity as its precursors.

In conclusion, the production of anti-*Candida* metabolites from *Streptomyces* HC14 is mainly associated with high concentrations of malt extract and glucose. GC–MS study combined with computational biology techniques has identified two putative metabolites as source of activity against *Candida albicans*: pyrrolo[1,2-a]pyrazine-1,4-dione, hexahydro-3-(phenylmethyl), and di-n-octyl phthalate. This expands our understanding of the range of antifungal compounds derived from *Streptomyces* and can serve as a foundation for further research into ligand-target interactions in *Candida albicans*.

## Data Availability

The datasets used and/or analyzed during the current study are available from the corresponding author on reasonable request.
